# Primary Pulmonary Leiomyosarcoma Successfully Treated With Radiotherapy Followed by Chemotherapy After Recanalization of Central Airway Obstruction by Bronchoscopic Cryotherapy: A Case Report

**DOI:** 10.7759/cureus.106168

**Published:** 2026-03-30

**Authors:** Masami Yamazoe, Kento Yasuda, Kohei Morikawa, Koki Kawahara, Kojiro Uemura

**Affiliations:** 1 Respiratory Medicine, Hakodate Municipal Hospital, Hakodate, JPN

**Keywords:** bronchoscopic cryotherapy, chemotherapy, doxorubicin, ifosfamide, primary pulmonary leiomyosarcoma, radiotherapy for local control

## Abstract

Primary pulmonary leiomyosarcoma (PPL) is the most common histological subtype of primary pulmonary sarcomas, although it is relatively rare. Herein, we report a case of a 76-year-old man with a mass arising in the hilar region of the right upper lobe of the lung and progressing to the right main bronchus, obstructing the bronchial lumen. He was admitted due to a five-day history of worsening respiratory distress and had a history of left upper lobectomy for primary lung adenocarcinoma. Bronchoscopic cryotherapy enabled the diagnosis of leiomyosarcoma of the lung and was effective in the recanalization of the right main bronchus obstruction caused by the tumor; it also improved his respiratory symptoms and hypoxia. Considering the risk of tumor invasion into the right main bronchus, the patient’s advanced age, and his medical history of left upper lobectomy, surgical resection was deemed extremely difficult. He was treated with radiotherapy for local control followed by doxorubicin and ifosfamide combination chemotherapy, with improvement of chest computed tomography and bronchoscopic findings. Twelve months have passed since the start of radiotherapy for local control, and he has completed the 10th cycle of combination chemotherapy without any signs of tumor recurrence. This case demonstrates that radiotherapy for local control followed by combination chemotherapy with doxorubicin and ifosfamide may improve the prognosis of patients with unresectable PPL.

## Introduction

Leiomyosarcoma is a relatively rare tumor, accounting for 17.4% of all sarcomas [1]. Leiomyosarcoma has been observed to originate from the smooth muscle cells in any location, but most often arises in the uterus, gastrointestinal tract, and soft tissues [2]. Primary pulmonary sarcomas account for less than 0.5% of all lung malignancies [3]. Primary pulmonary leiomyosarcoma (PPL) is the most common histological subtype of primary pulmonary sarcomas, accounting for around 30% of all primary pulmonary sarcoma cases [4]. Due to the rarity of PPL, there is limited consensus regarding the optimal treatment for this disease. Surgical resection is considered the best treatment for PPL, but the usefulness of radiotherapy or chemotherapy is uncertain [5]. Radiotherapy is generally reserved for local control in patients with incomplete resection, unresectable disease, or serious complications [5,6]. Chemotherapy is indicated for the advanced stage, as well as for patients with extrathoracic metastases [5].

Endobronchial cryotherapy is a treatment method that allows tissue to be frozen and removed from the bronchial lumen by direct application of an extremely cold cryoprobe [7]. Bronchoscopic cryotherapy (BC) is frequently used for biopsies, the extraction of endobronchial exophytic tumors, and the removal of blood clots and foreign bodies [7].

In this report, we present a case of an unresectable PPL that was successfully treated with recanalization of central airway obstruction with BC, followed by radiotherapy for local control, and then combination chemotherapy with doxorubicin and ifosfamide.

## Case presentation

**Figure 1:**
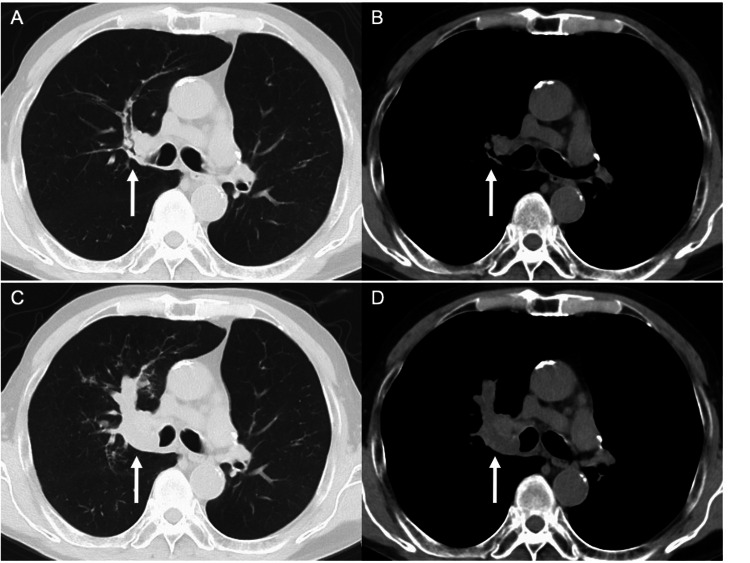
Chest computed tomography performed 14 months before admission shows thickening of the bronchial wall in the hilar region of the right upper lobe of the lung (white arrow) (A, B), which evolved into an irregular mass extending into the bronchial lumen of the right upper lobe (white arrow) two months before admission (C, D).

**Figure 2:**
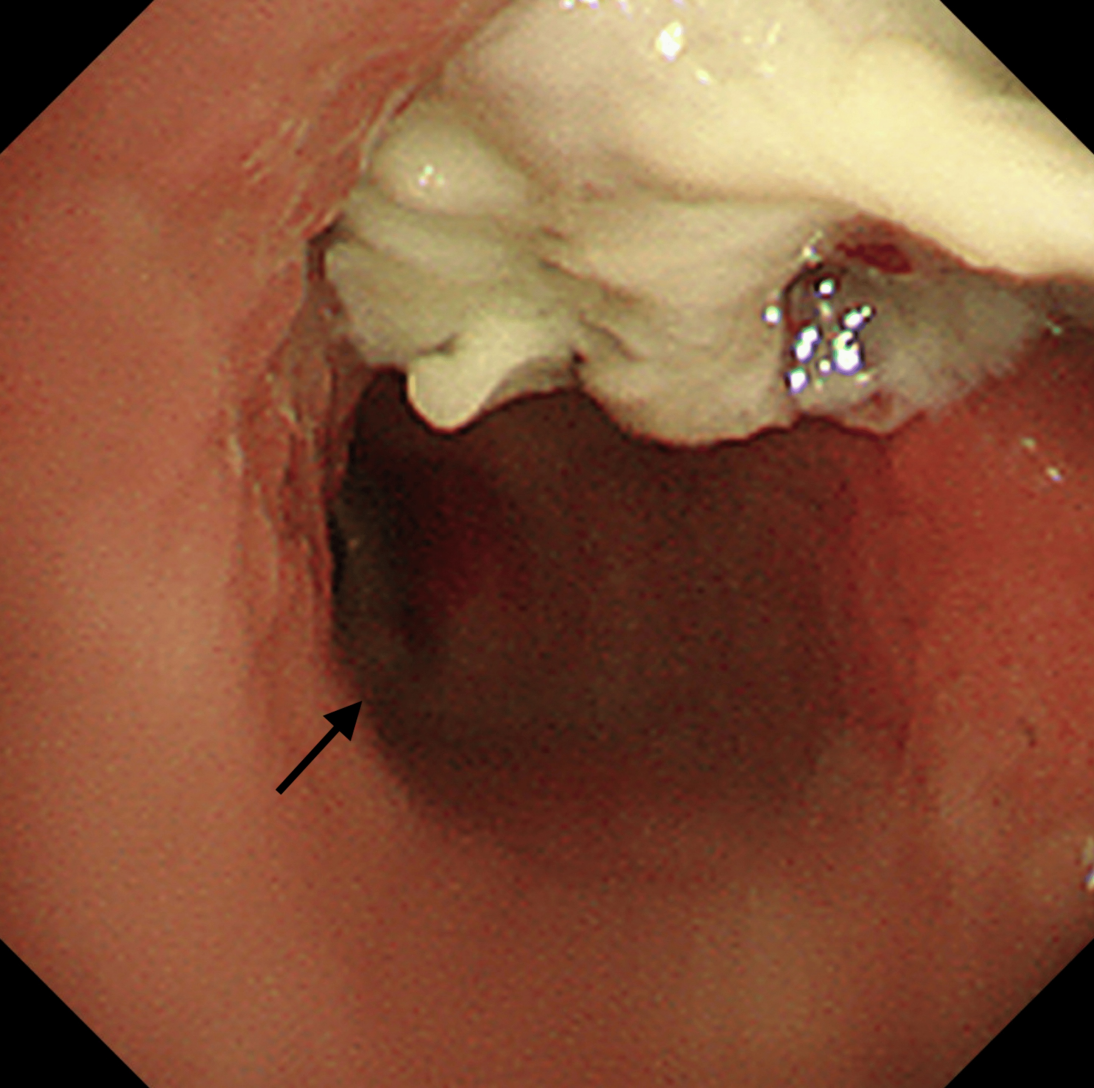
Bronchoscopy performed one month before admission shows the obstruction of the orifice of the right upper lobe bronchus by a mass covered with white material. The patency of the truncus intermedius was confirmed (black arrow).

A 76-year-old Japanese man underwent left upper lobectomy for primary lung adenocarcinoma in the left lung S^1+2^a (p-stage IA2, pT1bN0M0 under the TNM classification, eighth edition) in August 2021. He did not receive adjuvant chemotherapy and was monitored by imaging. He was an ex-smoker (one pack per day for 53 years). He had also been treated for bronchial asthma with inhaled corticosteroids and long-acting β_2_-agonists since 2009. His chest computed tomography (CT) in November 2023 showed thickening of the bronchial wall in the hilar region of the right upper lobe of the lung (Figures 1A, 1B), which evolved in November 2024 into an irregular mass extending into the bronchial lumen of the right upper lobe (Figures 1C, 1D). Although there were no particular symptoms, bronchoscopy was performed in December 2024. The orifice of the right upper lobe bronchus was obstructed by the mass covered with white material (Figure 2). A conventional forceps biopsy showed bronchial mucosa with inflammatory cell infiltration and necrotic tissue, but no malignant findings were observed. In January 2025, he was admitted to our hospital due to worsening respiratory distress that had lasted for five days.

On admission, a physical examination revealed the following: height, 165.0 cm; weight, 55.0 kg; temperature, 36.9 °C; blood pressure, 132/80 mmHg; heart rate, 86 beats/min; and oxygen saturation, 88% (on oxygen by nasal cannula at 3 L/min). On lung auscultation, breath sounds were decreased over the right lung. His blood tests on admission showed neutrophil-predominant leukocytosis, decreased hemoglobin levels, and elevated platelet counts. Renal function, liver function, and tumor marker (carcinoembryonic antigen and sialyl Lewis-x antigen) levels were within the normal ranges (Table 1).

**Table 1:** Quantitative laboratory investigations on admission

Test item	Result	Reference range
White blood cells	10.8 × 10^3^/μL	3.3–8.6
Neutrophils	94.2%	40.0-60.0
Hemoglobin	12.9 g/dL	13.7-16.8
Platelets	38.1 × 10^4^/μL	15.8-34.8
Aspartate transaminase (AST)	12 U/L	13-30
Alanine transaminase (ALT)	8 U/L	10-42
Blood urea nitrogen	17.5 mg/dL	8.0-20.0
Creatinine	0.76 mg/dL	0.65-1.07
C-reactive protein (CRP)	0.13 mg/dL	0.00-0.14
Carcinoembryonic antigen (CEA)	1.3 ng/mL	≤5.0
Sialyl Lewis-x antigen (SLX)	27 U/mL	≤38

**Figure 3:**
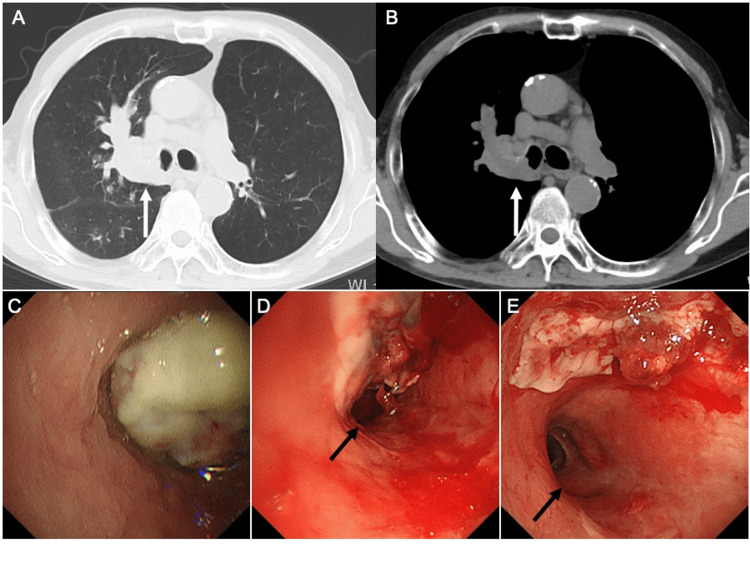
Chest computed tomography at admission shows the mass progressing to the right main bronchus, obstructing the bronchial lumen (white arrow) (A, B). Bronchoscopy before cryotherapy shows almost complete obstruction of the right main bronchus by a mass extending from the orifice of the right upper lobe bronchus (C), and following bronchoscopic cryotherapy, shows partial relief of the right main bronchus obstruction and patency of the truncus intermedius (black arrow) (D). Bronchoscopic image after repeat bronchoscopic cryotherapy, which was performed three days later, shows residual tumor protruding into the right main bronchus and patency of the truncus intermedius (black arrow) (E).

**Figure 4:**
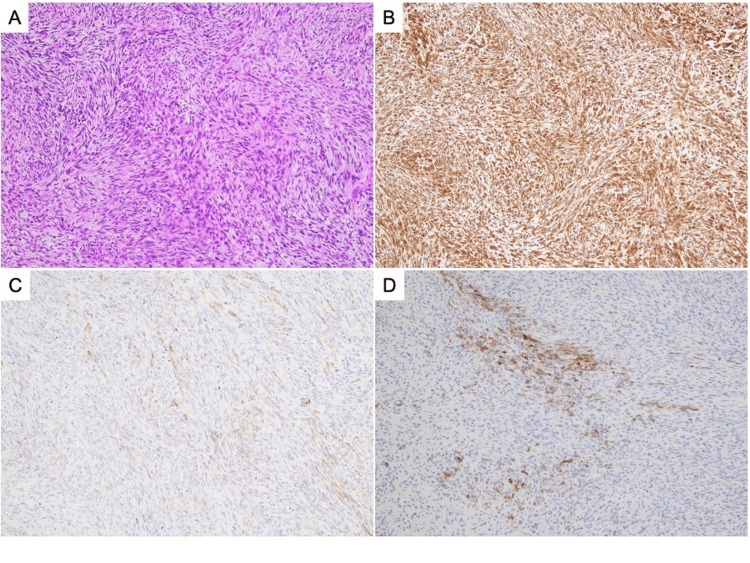
Hematoxylin and eosin staining of a cryobiopsy specimen shows densely packed, eosinophilic, spindle-shaped, tumor cells arranged in interweaving bundles (A, magnification x100). Additional pathological findings include a mitotic count of 18 mitoses per 10 high-power fields. Immunohistochemical staining shows positive findings for vimentin (B, magnification x100), smooth muscle actin (C, magnification x100), and h-caldesmon (D, magnification x100).

His chest CT showed that the mass had progressed to the right main bronchus, obstructing the bronchial lumen (Figures 3A, 3B). After oral intubation with an endotracheal tube (internal diameter 8.0 mm; Parker Flex-Tip PFHV-80; MC Medical, Inc., Tokyo, Japan) using a flexible bronchoscope (BF-P290; Olympus Corporation, Tokyo, Japan), bronchoscopy was performed. The right main bronchus was almost completely obstructed by the mass extending from the orifice of the right upper lobe bronchus (Figure 3C). The mass was detached from the membranous portion of the right main bronchus, allowing the bronchoscope to pass through, and the patency of the truncus intermedius was confirmed. During bronchoscopy, oxygen at 15 L/min was required due to cough and airway secretions, and it was determined that recanalization of the right main bronchus obstruction was necessary. After switching to another flexible bronchoscope (BF-1T260; Olympus Corporation), the mass was resected using a flexible cryoprobe (flexible, single-use, Φ1.7 mm cryoprobe; ERBE, Tübingen, Germany). The obstruction of the right main bronchus was partially relieved (Figure 3D), and his respiratory status improved promptly. Bronchoscopy was repeated three days later, and the remaining portion of the mass that had protruded into the right main bronchus was resected using a cryoprobe, as before (Figure 3E). Histological examination of the mass with hematoxylin and eosin staining showed densely packed, eosinophilic, spindle-shaped tumor cells arranged in interweaving bundles (Figure 4A). The mitotic count was 18 mitoses per 10 high-powered fields. The MIB-1 labeling index was 62.5%. Immunohistochemical staining was positive for vimentin, smooth muscle actin, and h-caldesmon (Figures 4B-4D). Immunohistochemical staining was negative for AE1/AE3, CK5/6, CK7, CK20, p40, p53, S100, and CD31. The pathological morphology and immunohistochemical profile were most consistent with grade 3 leiomyosarcoma according to the French Federation of Cancer Centres Sarcoma Group (FNCLCC) system. ^18^F-fluorodeoxyglucose (FDG)-positron emission tomography (PET)/computed tomography (CT), which was performed before admission, showed high FDG accumulation in the mass, with a maximal standardized uptake value of 13.51, and no other areas of abnormal FDG accumulation. Brain magnetic resonance imaging (MRI) did not show any signs of a mass effect or pathological intracranial enhancement. The patient was diagnosed with PPL.

**Figure 5:**
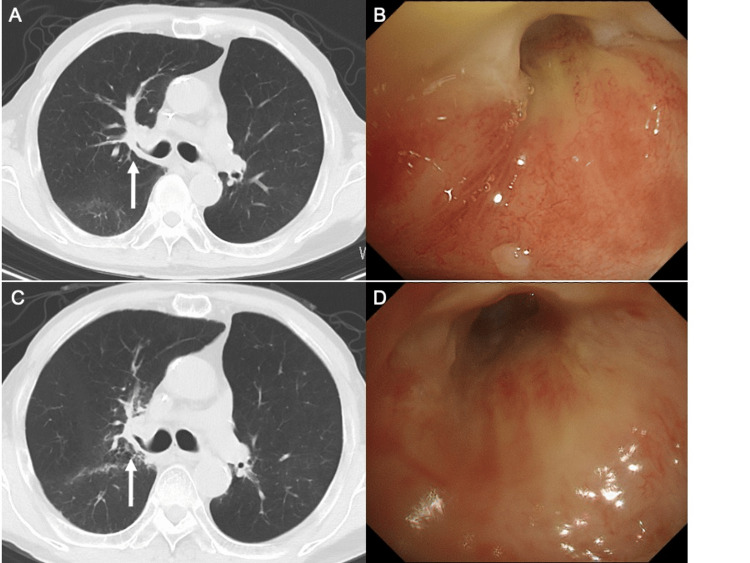
Chest computed tomography shows tumor shrinkage (white arrow) equivalent to a partial response at the end of the fourth cycle of combination chemotherapy (A), with tumor shrinkage (white arrow) maintained at the end of the eighth cycle of combination chemotherapy (C). Bronchoscopic image shows patency of the orifice of the right upper lobe bronchus at the end of the fourth cycle of combination chemotherapy (B), and slight improvement of patency at the end of the eighth cycle of combination chemotherapy (D).

Complete resection of the tumor was considered to be very difficult due to the risk of tumor invasion into the right main bronchus. Furthermore, given his advanced age and medical history of left upper lobectomy, it was decided that he was ineligible for surgical resection. Since there was concern about the right main bronchus obstruction due to tumor recurrence, he was initially started on radiotherapy for local control the day after the second BC. Simulation was performed using non-contrast CT in the supine position with both arms raised above the head under free-breathing conditions. The gross tumor volume (GTV) was defined as the clinically and radiographically evident tumor based on chest CT, FDG-PET/CT, and bronchoscopic findings. The clinical target volume (CTV) was generated by adding a 5-mm margin to the GTV to account for potential microscopic disease extension. The planning target volume (PTV) was created by adding a 5-mm setup margin to the CTV. Respiratory motion was managed under free-breathing conditions. Treatment planning was performed using intensity-modulated radiotherapy (IMRT) based on three-dimensional CT images. A total dose of 45 Gy in 15 fractions once daily, 5 times per week, was prescribed. The planning objective was to ensure that 80% of the PTV received 100% of the prescribed dose, which was achieved. Two weeks after completing radiotherapy, combination chemotherapy with doxorubicin (75% of 20 mg/m^2^ body surface area for three days) and ifosfamide (75% of 2000 mg/m^2^ body surface area for five days), followed by pegfilgrastim (3.6 mg subcutaneously, day 6), was started while taking into account the risk of adverse events. However, due to the development of febrile neutropenia (grade 4 Common Terminology Criteria for Adverse Events (CTCAE) v5), the doses of doxorubicin and ifosfamide were changed to 65% from the second cycle of combination chemotherapy. Combination chemotherapy was repeated every four or five weeks. He received four cycles of combination chemotherapy after radiotherapy for local control, which resulted in tumor shrinkage equivalent to a partial response on CT (Figure 5A) and patency of the orifice of the right upper lobe bronchus on bronchoscopy (Figure 5B). He continued to receive combination chemotherapy, and tumor shrinkage (Figure 5C) and patency (Figure 5D) were maintained even after the end of the eighth cycle of combination chemotherapy. No adverse events occurred during combination chemotherapy except for anemia (grade 2 CTCAE v5) and thrombocytopenia (grade 2 CTCAE v5). Twelve months have passed since the start of radiotherapy for local control, and he has completed the 10th cycle of combination chemotherapy without any signs of tumor recurrence. His Eastern Cooperative Oncology Group (ECOG) performance status was 1, the same as when he was started on radiotherapy for local control.

## Discussion

PPL appears to originate from the smooth muscle cells of the bronchial structures and pulmonary blood vessels [2] and is classified into three types: intraluminal type, which arises in the central bronchi and grows within the bronchial lumen; pulmonary vascular type, which originates from the larger pulmonary artery and grows along the vascular wall; and intrapulmonary type, which arises in peripheral lung tissue and progresses to the pulmonary parenchyma [8-11]. PPL can present with a broad range of nonspecific symptoms, including chest pain, dyspnea, hemoptysis, and cough, but it can also be asymptomatic. As with primary lung cancer, chest symptoms may depend on the location of the tumor [4]. Radiological findings are also diverse and nonspecific, ranging from collapse, consolidation, or a solitary mass to bilateral nodular shadows or patchy infiltrates, making them difficult to distinguish from primary lung cancer [5]. Furthermore, because PPL has a lower tendency to exfoliate than epithelial tumors, bronchoscopic procedures, including conventional forceps biopsy, lavage, and brushing, are often nondiagnostic, and excision biopsy is required for diagnosis [2,6]. Given that PPL is a rare disease, the diagnosis can be challenging. In the present case, there were no symptoms at the time of bronchoscopy performed one month before admission, and allergic bronchopulmonary aspergillosis (ABPA) was initially suspected based on the patient’s medical history of bronchial asthma and chest CT findings. Then, bronchoscopy showed a mass covered with white material, and a conventional forceps biopsy failed to yield a diagnosis. Primary lung cancer was suspected, and transbronchial cryobiopsy was being considered as an alternative. However, one month later, the tumor protruded into the right main bronchus, obstructing the bronchial lumen and causing respiratory distress and hypoxia in the patient. This case highlights the need for clinicians to consider rare tumors such as PPL when evaluating pulmonary masses growing within the bronchial lumen.

A retrospective cohort study of 231 PPL patients showed that the median overall survival (OS) for this disease was 14.0 months, and the one-, three-, and five-year OS values were 52.7%, 29.0%, and 22.2%, respectively [6]. In particular, the median OS for inoperable cases was only 4 months, and even for surgical cases, it was only 33 months [6]. It has also been reported that advanced stage was an independent prognostic factor for PPL, whereas surgical resection was an independent protective factor [6]. Moran et al. showed that the median OS for high-grade PPL, which was characterized by high cellularity, marked pleomorphism and atypia, frequent areas of hemorrhage and necrosis, and high mitotic activity, was approximately five months [12]. Hasegawa suggested that the histological grading of soft tissue sarcomas based on the MIB-1 labeling index was useful for predicting prognosis and was advantageous in terms of objectivity and reproducibility compared to the FNCLCC system [13]. In the present case, the MIB-1 labeling index was high at 62.5%, and the tumor was classified in the high-grade (grade 3) category, the same grade as in the FNCLCC system. This grade suggested a poor prognosis [13].

A multidisciplinary approach, including surgery, radiotherapy, and chemotherapy, is important in the treatment of PPL. Surgical resection is considered the best treatment for PPL [5]. However, in the present case, surgical resection was considered extremely difficult, given the risk of tumor invasion into the right main bronchus, his advanced age, and his medical history of left upper lobectomy. Radiotherapy or chemotherapy is recommended in cases of incomplete resection, unresectable tumors, and patients with histologically high-grade malignancy, but the usefulness of either therapy is unclear [2,6]. According to a propensity score matching analysis between a group that received radiotherapy and a group that did not, based on factors such as age, gender, ethnicity, married status, pathological differentiation, summary stage, laterality, primary site, lymph node metastases, distant metastases, whether or not surgery was performed, and surgery type, radiotherapy has not been shown to provide any benefit in terms of OS in patients with PPL [6]. However, Krishnan et al. reported a case where radiotherapy (45 Gy in 15 fractions) was effective in local tumor control [14]. No effective chemotherapy regimens have been established for patients with advanced or metastatic PPL. Judson et al. reported the results of a phase 3, randomized controlled trial in which combination chemotherapy with doxorubicin and ifosfamide as first-line treatment produced a response rate (RR) of 26% in patients with locally advanced, unresectable, or metastatic high-grade soft tissue sarcomas, including leiomyosarcoma [15]. In contrast, a retrospective study examining the efficacy of doxorubicin and ifosfamide combination therapy as first-line treatment in 51 patients with metastatic or recurrent leiomyosarcomas found an overall RR of 12%, with a higher rate in patients with uterine leiomyosarcoma (17%) than in those with nonuterine leiomyosarcoma (5%) [16]. Pazopanib and trabectedin are also approved for the treatment of patients with recurrent or metastatic leiomyosarcoma who have failed anthracycline-based chemotherapy [16]. In particular, pazopanib, a multi-tyrosine kinase inhibitor, has shown modest efficacy in soft tissue sarcomas, and it was reported that pazopanib helped to maintain stable disease and prolong survival in an elderly patient with PPL [14]. Eribulin has also shown efficacy in patients with advanced liposarcoma or leiomyosarcoma who have received two or more prior chemotherapy regimens [17]. The present case suggests that local control of PPL with radiotherapy followed by combination chemotherapy with doxorubicin and ifosfamide may lead to prolonged survival even in an unresectable case of PPL.

A specific technique that enables rapid debulking of the occluded airways by a cryoprobe is cryorecanalization [18], which is an efficient and relatively safe intervention for patients with central airway obstruction [7]. The most common complication of BC is endobronchial hemorrhage [7]. In the present case, the conventional forceps biopsy was nondiagnostic, but transbronchial cryobiopsy enabled the diagnosis of PPL. The patient’s symptoms and hypoxia were also improved by recanalization of the right main bronchus obstruction with BC. BC-related endobronchial hemorrhage was mild and was stopped by the instillation of epinephrine solution (1 mg/100 mL normal saline). Although preoperative diagnosis of PPL with bronchoscopic examinations, including conventional forceps biopsy, lavage, and brushing, may be challenging [2,6], transbronchial cryobiopsy may be a useful and safe technique for obtaining a histological diagnosis.

## Conclusions

In this report, we discussed a case of unresectable PPL that was successfully treated with radiotherapy for local control followed by combination chemotherapy with doxorubicin and ifosfamide. BC was effective in diagnosing PPL and recanalizing the central airway obstruction caused by the tumor. Treatment algorithms for PPL have not been established. However, multidisciplinary treatment, such as radiotherapy for local control followed by chemotherapy, appears to be crucial to achieve long-term survival in patients with unresectable PPL while maintaining their quality of life. Further research on novel treatment combinations is needed to establish optimal treatment strategies for unresectable PPL.

## References

[1] Mack TM (1995). Sarcomas and other malignancies of soft tissue, retroperitoneum, peritoneum, pleura, heart, mediastinum, and spleen. Cancer.

[2] Xie X, Chen Y, Ding C (2016). Primary pulmonary leiomyosarcoma: a case report. Oncol Lett.

[3] Etienne-Mastroianni B, Falchero L, Chalabreysse L, Loire R, Ranchère D, Souquet PJ, Cordier JF (2002). Primary sarcomas of the lung: a clinicopathological study of 12 cases. Lung Cancer.

[4] Tanaka K, Iwata T, Nishii K (2019). A case of primary pulmonary leiomyosarcoma completely resected after neoadjuvant chemotherapy. Surg Case Rep.

[5] Sultana S, Thangaswamy D (2025). Pulmonary leiomyosarcoma: a diagnostic challenge. Cureus.

[6] Qin BD, Jiao XD, Zang YS (2018). Primary pulmonary leiomyosarcoma: a population-based study. Lung Cancer.

[7] Jeong JH, Kim J, Choi CM, Ji W (2023). Clinical outcomes of bronchoscopic cryotherapy for central airway obstruction in adults: an 11-years’ experience of a single center. J Korean Med Sci.

[8] Yu H, Ren H, Miao Q, Wang Z, Zhang Z, Xu L (1997). Pulmonary leiomyosarcoma. Chin Med Sci J.

[9] Yamada N, Kamei S, Yasuda F, Isaka N, Yada I, Nakano T (1998). Primary leiomyosarcoma of the pulmonary artery confirmed by catheter suction biopsy. Chest.

[10] Muscolino G, Bedini AV, Buffa PF (2000). Leiomyosarcoma of the bronchus: report of two cases of resection with long-term follow-up. J Thorac Cardiovasc Surg.

[11] Yata Y, Ito Y, Iwamoto K, Kumazawa A, Yosihara T, Kato T, Okazawa M (2019). A case of primary pulmonary leiomyosarcoma. Respir Med Case Rep.

[12] Moran CA, Suster S, Abbondanzo SL, Koss MN (1997). Primary leiomyosarcomas of the lung: a clinicopathologic and immunohistochemical study of 18 cases. Mod Pathol.

[13] Hasegawa T (2007). Histological grading and MIB-1 labeling index of soft-tissue sarcomas. Pathol Int.

[14] Krishnan S, Ramalingam VS, Patel C, Heisick J (2025). Primary pulmonary leiomyosarcoma: a case of prolonged survival with radiotherapy and pazopanib in a 90-year-old female. Respirol Case Rep.

[15] Judson I, Verweij J, Gelderblom H (2014). Doxorubicin alone versus intensified doxorubicin plus ifosfamide for first-line treatment of advanced or metastatic soft-tissue sarcoma: a randomised controlled phase 3 trial. Lancet Oncol.

[16] Akin S, Dizdar O, Karakas Y, Turker A, Kars A (2018). Ifosfamide and doxorubicin in the treatment of advanced leiomyosarcoma. Curr Probl Cancer.

[17] Schöffski P, Chawla S, Maki RG (2016). Eribulin versus dacarbazine in previously treated patients with advanced liposarcoma or leiomyosarcoma: a randomised, open-label, multicentre, phase 3 trial. Lancet.

[18] Hetzel M, Hetzel J, Schumann C, Marx N, Babiak A (2004). Cryorecanalization: a new approach for the immediate management of acute airway obstruction. J Thorac Cardiovasc Surg.

